# Eccrine Poroma and Trichoblastoma Concurrently Arising Within a Nevus Sebaceous of Jadassohn: A Case Report

**DOI:** 10.7759/cureus.95544

**Published:** 2025-10-27

**Authors:** Anahi Santana Barreto, Amara Hazel Solorio Rivera, Nayeli González Pérez, María Fernanda Limón Limón, Ernesto Velazco Manzo

**Affiliations:** 1 Dermatology, Instituto Dermatológico de Jalisco "Dr. José Barba Rubio", Zapopan, MEX; 2 Dermatologic Surgery, Instituto Dermatológico de Jalisco “Dr. José Barba Rubio”, Zapopan, MEX

**Keywords:** adnexal tumor, eccrine poroma, nevus sebaceous, sebaceous nevus of jadassohn, trichoblastoma

## Abstract

Nevus sebaceous of Jadassohn (NSJ) is a congenital, benign hamartomatous lesion characterized by a well-demarcated, hairless, yellowish plaque, usually located on the scalp or face. In adolescence or adulthood, benign or malignant secondary tumors may develop within the nevus sebaceous. The most frequently associated benign tumors include trichoblastoma and syringocystadenoma papilliferum. The presence of an eccrine poroma within a nevus sebaceous is extremely rare, with only six cases documented in the literature. We present a case of a 33-year-old woman with a long-standing alopecic plaque on the scalp that progressively developed into two nodular growths. Histopathological examination of the biopsy revealed features compatible with a sebaceous nevus associated with an eccrine poroma and trichoblastoma. Complete surgical excision of the sebaceous nevus, including both neoplasms, was performed.

This study represents one of the few reported cases of an eccrine poroma arising within a nevus sebaceous, underscoring the importance of surveillance and timely excision when indicated.

## Introduction

Nevus sebaceous of Jadassohn (NSJ) is a benign hamartomatous lesion composed of epidermal, follicular, sebaceous, and both apocrine and eccrine glandular structures. Clinically, it is present at birth as a well-demarcated, alopecic, yellowish to skin-colored plaque, most commonly located on the scalp. During puberty, it may grow thicker and acquire a verrucous appearance, influenced by androgenic activity, which justifies continued clinical monitoring [1].

Up to 15% of NSJ cases develop secondary tumors during adolescence or adulthood. Of these, 90.3% are benign, with syringocystadenoma papilliferum and trichoblastoma being the most common. Although basal cell carcinoma (BCC) has historically been reported as the most frequent malignant tumor, contemporary series with dermatopathological review indicate that a considerable proportion of these cases were actually trichoblastomas misclassified as BCC due to histological overlap. Consequently, the true incidence of BCC in NSJ appears to be low (≈1%). From a molecular perspective, NSJ and its associated adnexal neoplasms (e.g., trichoblastoma) frequently harbor postzygotic activating mutations in HRAS/KRAS, whereas BCC is more commonly driven by alterations in the hedgehog signaling pathway (e.g., PTCH1, SMO) [2-4].

Eccrine poroma is a benign adnexal neoplasm originating in the intraepidermal portion of the sweat gland duct. Although it typically appears on the palms and soles, it may also occur in other areas rich in eccrine glands, such as the scalp [1,4]. ​​In the present case, histopathological examination confirmed the diagnosis of eccrine poroma and a trichoblastoma arising within a nevus sebaceous of Jadassohn, a rare combination.

## Case presentation

**Figure 1:**
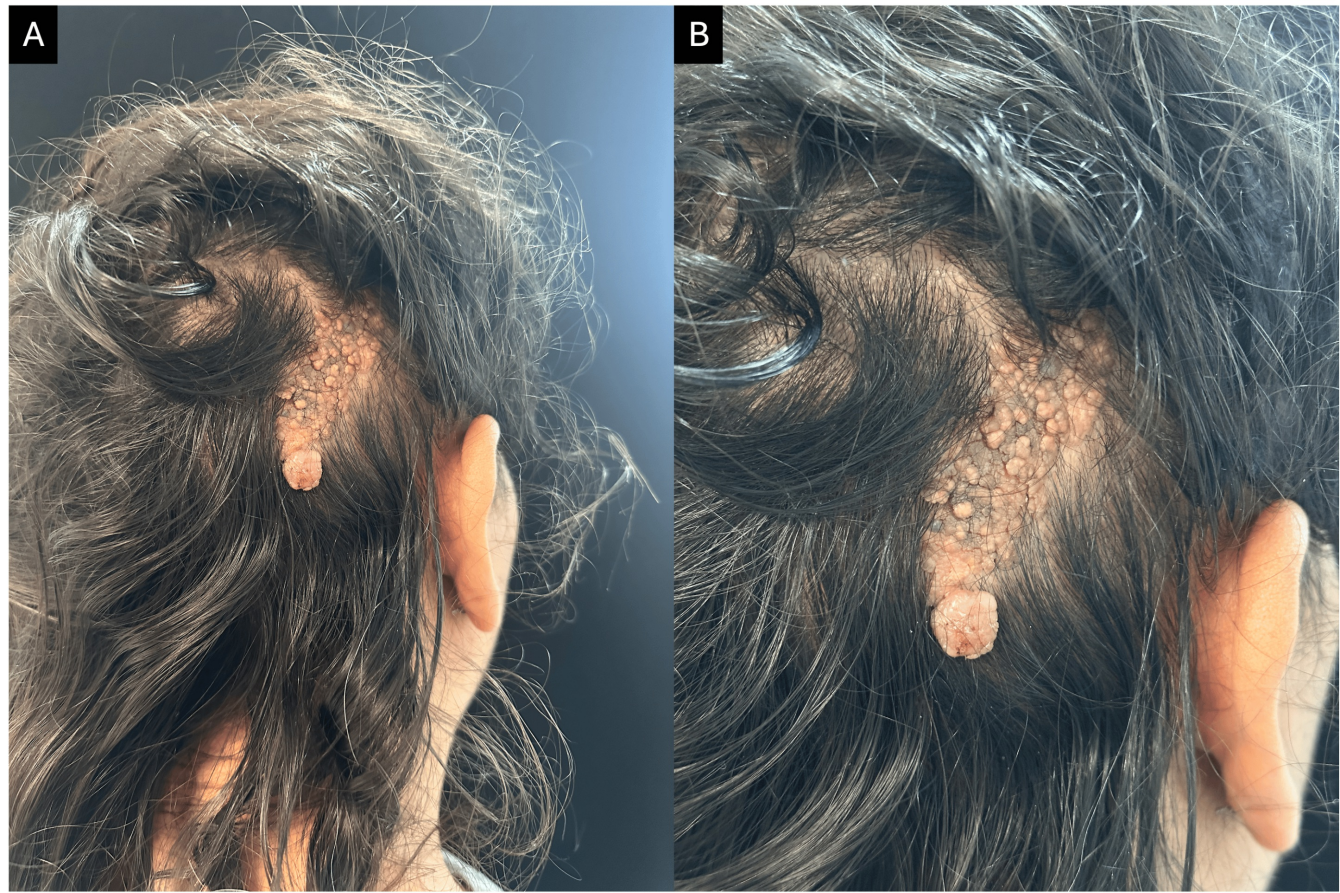
Clinical presentation of the nodular lesion arising from a verrucous plaque on the scalp. (A and B) A solitary pink nodular lesion with a verrucous surface, measuring 1 × 1 × 1.2 cm, arising on a yellowish, verrucous plaque measuring 6 × 2.5 × 0.2 cm, located on the right parietal region of the scalp.

A 33-year-old female patient with no significant medical history presented to the Dermatology Department with a nodule that had enlarged over the past four years, arising within a congenital alopecic plaque. Physical examination revealed a 6 x 2.5 x 0.2 cm, pink to brown, verrucous plaque with sharp borders. Also, a 1.2 x 1 x 1 cm, pink, multilobulated, pedunculated nodule with a firm consistency was found at the lower end of the plaque (Figures 1A, 1B).

**Figure 2:**
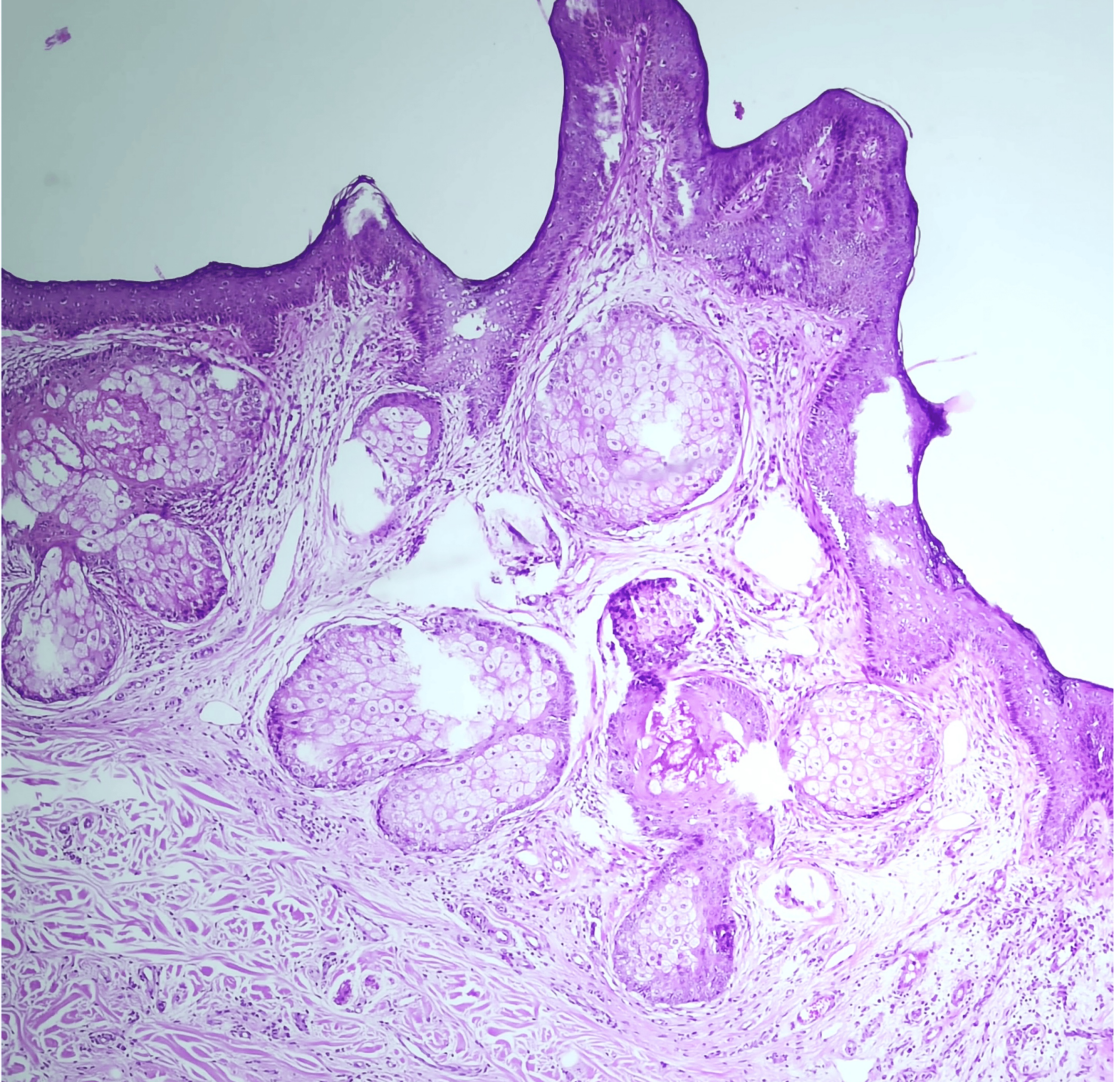
Epidermal papillomatosis associated with increased sebaceous glands and abortive hair follicles within the dermis, consistent with nevus sebaceous (H&E, 10×).

**Figure 3:**
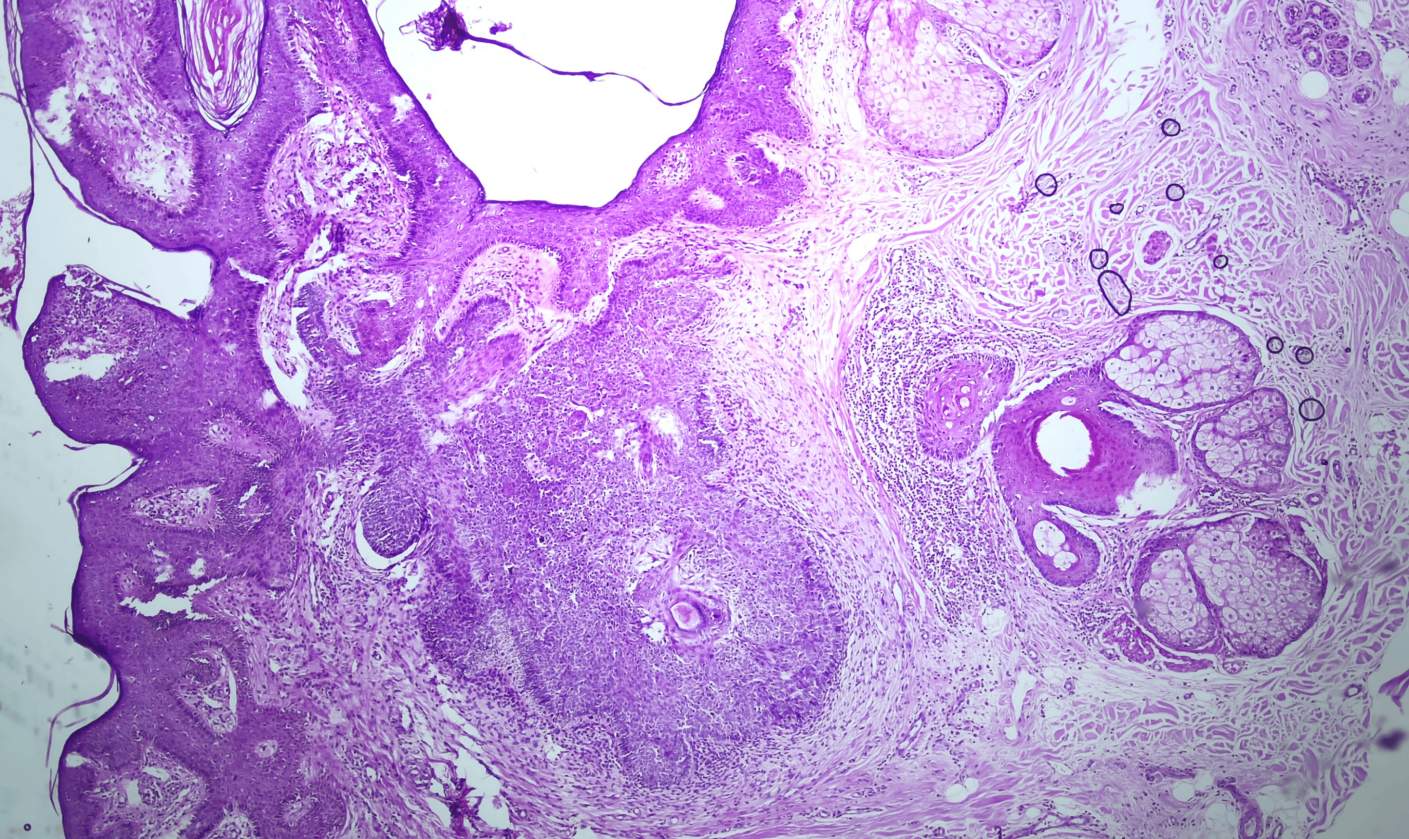
Follicular proliferation composed of basaloid cells with peripheral palisading (H&E, 10×).

A skin biopsy was obtained from both the verrucous plaque and the nodular lesion. The plaque biopsy demonstrated epidermal papillomatosis with acanthosis associated with an increase in sebaceous glands and abortive hair follicles, consistent with nevus sebaceous (Figure 2). Additionally, a follicular proliferation composed of basaloid cells with palisading cells at the periphery was also observed, consistent with trichoblastoma (Figure 3).

**Figure 4:**
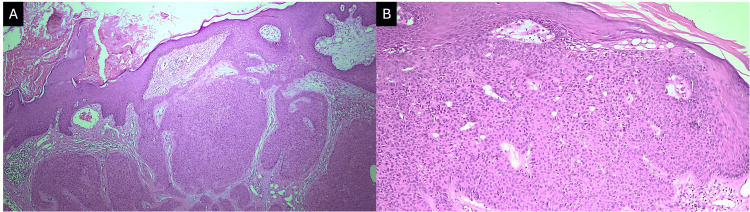
Histopathological features of the nodular lesion consistent with eccrine poroma. (A) Proliferation of uniform small cuboidal cells arranged in anastomosing bands, with visible ductal lumina, embedded in a fibrovascular stroma (H&E, 10×). (B) Higher magnification showing ductal differentiation and poroid cells with scant cytoplasm and round nuclei (H&E, 40×).

**Figure 5:**
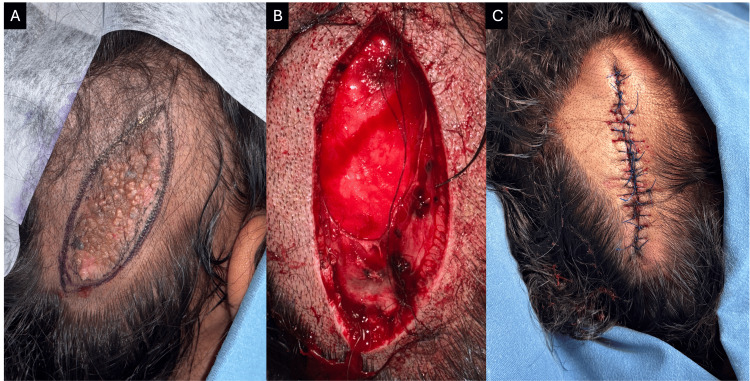
Intraoperative and postoperative views of surgical excision of the lesion. (A-C) Intraoperative view showing complete surgical excision of both the verrucous plaque and the nodular lesion in a single specimen, with clear margins achieved.

The biopsy of the nodular lesion revealed a proliferation of small cuboidal basaloid cells with ductal differentiation and anastomosing bands within a fibrovascular stroma, consistent with eccrine poroma (Figures 4A, 4B). Based on the clinical and histopathological findings, a diagnosis of eccrine poroma and trichoblastoma arising within a nevus sebaceous was established. Complete surgical excision of the lesions was performed (Figure 5A-5C). At the three-month follow-up, there was no evidence of recurrence.

## Discussion

​​Nevus sebaceous, also known as nevus sebaceous of Jadassohn or organoid nevus, is a hamartomatous lesion. It is usually present at birth in the scalp as a well-defined, alopecic plaque, which may be yellow-orange or darker. Over time, especially during puberty, it tends to become more elevated and verrucous [5].

​​Nevus sebaceous is considered to be caused by postzygotic mosaic mutations in the HRAS or KRAS genes, leading to somatic mosaicism and activation of pathways such as MAPK and PI3K-AKT, promoting cellular proliferation. These mutations may also be present in secondary tumors, suggesting a common origin; however, additional mutations, such as those in TP53 and NOTCH2, have been described and are associated with tumor progression. Other syndromes that belong to the group of mosaic RASopathies include the sebaceous nevus syndromes, which comprise Schimmelpenning syndrome, phacomatosis pigmentokeratotica, and cutaneous skeletal hypophosphatemia syndrome [3].

This lesion has the potential to develop into other tumors, most of which are benign. The two most frequently reported neoplasms are syringocystadenoma papilliferum and trichoblastoma, whereas eccrine poroma is exceptionally rare, with only a few cases described in the literature [5]. In a study of 706 patients, 21.4% developed secondary neoplasms; of these, 18.9% were benign and 2.5% malignant. Among the benign neoplasms, trichoblastoma was the most common (7.4%), followed by syringocystadenoma papilliferum (5.2%). Among the malignant neoplasms, basal cell carcinoma accounted for 1.1%, followed by squamous cell carcinoma with 0.57% [5].

Eccrine poroma is a benign adnexal neoplasm that originates from the intraepidermal portion of the eccrine sweat duct [6]. The most commonly affected sites are the palms and soles, while involvement of the scalp is rare [4,6]. Its association with NSJ is extremely rare, with only six cases documented to date [1,4]. A summary of previously reported cases of poroma arising within nevus sebaceous has been compiled from previously published reports and is presented in Table 1 [1,7-11].

**Table 1:** Reported cases of poroma arising in nevus sebaceous.

Studies	Year	Sex/age (years)	Location	Size of NS	Type	Evolution time	Clinical presentation of poroma	Other coexisting secondary neoplasms
Jaqueti et al.	2000	-	-	-	Apocrine poroma	-	-	-
Seo et al.	2004	Female/11	Scalp	2 × 2 cm	Apocrine poroma	1 year	Erythematous papule 0.3 × 0.3 cm	Tubular apocrine adenoma
Lee et al.	2009	Male/63	Left cheek	10 × 1.5 cm	Eccrine poroma	40 years	Multiple papules and 'pebble-like' nodules of varied size and color	Sebaceous adenoma, basal cell epithelioma
Wang et al.	2013	Female/48	Scalp	11 × 2.5 cm	Apocrine poroma	6 months	Red plaque of 2 cm in diameter	Trichoblastoma, sebaceous carcinoma
Cicek et al.	2015	Male/40	Scalp	3.5 × 1.9 cm	Eccrine poroma	Recent	Brown lesion with rough surface	Basal cell carcinoma
Girdwichai et al.	2016	Female/30	Scalp	3 × 6 cm	Eccrine poroma	8 months	Solitary erythematous nodule, slightly verrucous, 3 cm in diameter	None

The diagnosis is based on the clinical presentation and is confirmed by histopathological examination, which typically shows epidermal hyperplasia, immature hair follicles, and sebaceous and sweat gland components. When a secondary lesion is suspected, each lesion should be biopsied to confirm the diagnosis. Alternatively, an excisional biopsy of the entire lesion can be performed. In cases where a syndromic presentation is suspected, additional clinical evaluation and studies should be undertaken [12].

Complete surgical resection is the treatment of choice when excision is indicated. The decision to excise should be individualized, taking into account the patient’s age, the extent and location of the lesion, cosmetic considerations, and the presence of secondary tumors within the plaque. The risk of malignant transformation is low; therefore, an expectant management approach with regular follow-up may also be appropriate [5].

## Conclusions

Eccrine poroma arising within a nevus sebaceus is an exceptionally rare finding, and its coexistence with trichoblastoma makes this case even more unusual. This report underscores not only the importance of long-term clinical surveillance and timely histopathological evaluation of new growths within these lesions, but also the educational value of documenting such a rare coexistence. While complete surgical excision remains the treatment of choice when secondary tumors are present, conservative monitoring may also be appropriate in selected cases, reflecting the ongoing debate regarding optimal management.

## References

[1] Girdwichai N, Chanprapaph K, Vachiramon V (2016). Eccrine poroma arising within nevus sebaceous. Case Rep Dermatol.

[2] Ye Q, Wu Q, Jia M, Li FZ, Fang S (2023). Secondary tumors arising from nevus sebaceus: a multicenter collaborative study and literature review. Dermatology.

[3] Minowa T, Kamiya T, Hida T (2021). Genetic analyses of a secondary poroma and trichoblastoma in a HRAS-mutated sebaceous nevus. J Dermatol.

[4] Seo JK, Shin MK, Jeong KH, Lee MH (2020). Eccrine poroma arising within nevus sebaceous. Ann Dermatol.

[5] Idriss MH, Elston DM (2014). Secondary neoplasms associated with nevus sebaceus of Jadassohn: a study of 707 cases. J Am Acad Dermatol.

[6] Li J, Ding Y, Zhang S, He W (2023). Eccrine poroma: a case report. Asian J Surg.

[7] Jaqueti G, Requena L, Sánchez Yus E (2000). Trichoblastoma is the most common neoplasm developed in nevus sebaceus of Jadassohn: a clinicopathologic study of a series of 155 cases. Am J Dermatopathol.

[8] Seo YJ, Kim SS, Kim KH (2004). A case of nevus sebaceus associated with tubular apocrine adenoma and apocrine poroma. Korean J Dermatol.

[9] Lee GH, Kim DH, Lee SH (2009). A case of nevus sebaceus associated with sebaceous adenoma, basal cell epithelioma, and eccrine poroma. Korean J Dermatol.

[10] Wang E, Lee JS, Kazakov DV (2013). A rare combination of sebaceoma with carcinomatous change (sebaceous carcinoma), trichoblastoma, and poroma arising from a nevus sebaceus. J Cutan Pathol.

[11] Cicek AF, Aykan A, Yapici A (2015). Nevus sebaceus with basal cell carcinoma, poroma, and verruca vulgaris. Indian J Pathol Microbiol.

[12] Mitchell DC, Kuehn GJ, Scott GA, Doerr TD, Tausk F (2021). A rare case of porocarcinoma and trichoblastoma arising in a nevus sebaceus of Jadassohn. Case Rep Dermatol Med.

